# Population genetic structure of the *Culex pipiens* (Diptera: Culicidae) complex, vectors of West Nile virus, in five habitats

**DOI:** 10.1186/s13071-017-2594-6

**Published:** 2018-01-04

**Authors:** Andrea L. Joyce, Etienne Melese, Phuong-Thao Ha, Allan Inman

**Affiliations:** 1 Public Health, University of California Merced, 5200 North Lake Road, Merced, CA 95343 USA; 2 Department of Microbiology and Immunology, Life Sciences Institute, University of British Columbia, Vancouver, BC V6T 1Z3 Canada; 3 Santa Clara County Vector Control District, 1580 Berger Dr, San Jose, CA 95112 USA; 4 Merced County Mosquito Abatement District, 3478 Beachwood Dr, Merced, CA 95348 USA

**Keywords:** AFLPs, *Culex pipiens*, *Culex quinquefasciatus*, *Culex pipiens molestus*, Hybrids

## Abstract

**Background:**

The *Culex pipiens* complex consists of several morphologically similar, closely related species. In the United States, *Cx. pipiens* L. is distributed North of 39° latitude, while *Cx. quinquefasciatus* Say occurs South of 36° latitude; a hybrid zone occurs between these two latitudes including in the Central Valley of California. Members of the *Cx. pipiens* complex and their hybrids are vectors for West Nile virus (WNv). Hybrid offspring of *Cx. pipiens* and *Cx. quinquefasciatus* have been found to have enhanced transmission rates of WNv over those of pure populations of each species. We investigated whether hybrids of *Cx. pipiens* and *Cx. quinquefasciatus* occurred more frequently in any of five habitats which were dairies, rural, suburban, and urban areas, and wetlands. In addition, the proportion of alleles unique to *Cx. quinquefasciatus* and *Cx. pipiens* found in each habitat-associated population were determined.

**Methods:**

Amplified fragment length polymorphism (AFLP) markers were used to compare the population structure of the *Cx. pipiens* complex from each habitat to geographically distant populations considered pure *Cx. pipiens* and *Cx. quinquefasciatus*. Structure analyses were used to assign individuals to either *Cx. pipiens*, *Cx. quinquefasciatus*, or hybrids of the *Cx. pipiens* complex. The ancestry of hybrids (F1, F2, or backcrossed) in relation to the two parent populations was estimated for each Central Valley population. Loci unique to the pure *Cx. pipiens* population and the pure *Cx. quinquefasciatus* population were determined. The proportion of loci unique to *Cx. pipiens* and *Cx. quinquefasciatus* populations were subsequently determined for each population from the five Merced habitats and from the Oroville California population. The unique loci found in Merced populations and not in *Cx. pipiens* or *Cx. quinquefasciatus* were also determined. A principal components analysis was run, as was an analysis to determine loci under putative selection.

**Results:**

The Structure Harvester analysis found K = 3, and the *Culex pipiens* complex mosquitoes formed a genetic cluster distinct from *Cx. quinquefasciatus* and *Cx. pipiens*. Individuals collected from each habitat were nearly all hybrids. However, *Cx. pipiens* complex collected near dairies had more individuals categorized as *Cx. pipiens* than collections from the other habitats. None of the mosquitoes collected in Merced or Oroville were considered pure *Cx. quinquefasciatus*. Significant genetic divergence was detected among the *Cx. pipiens* complex from the five habitats in Merced; *Cx. pipiens* complex mosquitoes from dairies were divergent from the urban and suburban populations. New Hybrids analysis found that individuals from all five Merced habitat-associated populations and the population from Oroville were primarily categorized as hybrids backcrossed to the *Cx. pipiens* population. Finally, all five habitat-associated populations shared more alleles with *Cx. pipiens* than with *Cx. quinquefasciatus,* even though the pure *Cx. quinquefasciatus* population was more geographically proximate to Merced. Results from the principal component analysis, and the occurrence of several unique loci in Merced populations, suggest that *Cx. pipiens molestus* may also occur in the habitats sampled.

**Conclusions:**

Nearly all mosquitoes in the five habitats in Merced in the Central Valley of California area were hybrids of *Cx. pipiens* and *Cx. quinquefasciatus*, consisting of hybrids backcrossed to *Cx. pipiens.* Habitat-associated mosquitoes collected near dairies had more individuals consisting of pure *Cx. pipiens*, and no mosquitoes from Merced or Oroville CA classified as pure *Cx. quinquefasciatus*. The genetic distances among *Cx. pipiens* and *Cx. quinquefasciatus*, and hybrid populations agree with previous studies using other molecular markers. *Cx. pipiens* hybrids in Merced shared more alleles with *Cx. pipiens* than *Cx. quinquefasciatus* which was unexpected, since Merced is geographically closer to the northern limit of *Cx. quinquefasciatus* distribution. *Culex pipiens molestus* may occur in more habitats in the Central Valley than previously suspected, which warrants further investigation. Future studies could investigate the vector competence of hybrids backcrossed to either *Cx. pipiens* or *Cx. quinquefasciatus* parent for their ability to transmit West Nile virus.

**Electronic supplementary material:**

The online version of this article (10.1186/s13071-017-2594-6) contains supplementary material, which is available to authorized users.

## Background

The *Culex pipiens* species complex consists of several morphologically similar closely related mosquito species involved in the transmission of West Nile virus [1]. West Nile virus (WNv), originally described from Uganda and introduced on the East Coast of the United States in 1999, spread rapidly across North America and reached the West Coast in several years. West Nile virus is enzootic, primarily contained in a bird-mosquito transmission cycle with humans being incidental hosts, although prevention of human infection with West Nile remains a public health concern where the virus has been introduced [2].

**Fig. 1:**
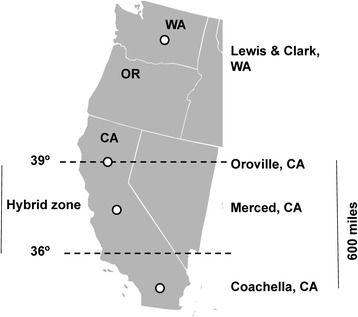
Map of the Pacific Coast of the United States, showing the 39° latitude southern boundary of the distribution of *Cx. pipiens* and the 36° latitude northern boundary of *Cx. quinquefasciatus*. The zone between the two latitudes (between 39° and 36°) is the hybrid zone for the two *Culex* species. Collections from this study are *Cx. pipiens* from Lewis and Clark Reservoir Washington, *Cx. pipiens* complex from Oroville California and Merced County California, and *Cx. quinquefasciatus* from Coachella California. Merced collections are further detailed in Table 1 and Fig. 2

In the United States, *Culex pipiens* L. and *Cx. quinquefasciatus* Say are introduced species, with *Cx. pipiens* generally found North of 39° latitude, and *Cx. quinquefasciatus* South of 36° latitude; the region between these latitudes which includes the Central Valley of California is a contact zone where hybrids of *Cx. pipiens* and *Cx. quinquefasciatus* occur [3–6] (Fig. 1). One ecological factor in California which contributes to hybrid formation and maintenance of the hybrid zone is that *Cx. pipiens* inhabits cooler northern latitudes and undergoes a winter reproductive diapause, while *Cx. quinquefasciatus* resides in more southern latitudes and can overwinter without diapausing. Both *Cx. pipiens* and *Cx. quinquefasciatus* are efficient vectors of WNv [7], but the two species differ in their host preferences. *Cx. pipiens* host feeding preference is primarily ornithopihlic [4, 8], and *Cx. quinquefasciatus* feeds on both birds and mammals, which can bridge the transmission of WNv between avian and mammalian hosts [9–11]. In addition, another very closely related member of the complex, *Culex pipiens molestus*, is reported in some areas of the United States, and typically inhabits primarily underground areas such as sewers and basements [12, 13], while in Europe *Cx. pipiens molestus* hybrids have been found underground as well as aboveground in animal shelters [8, 14].

The morphological identification and separation of members of the *Culex pipiens* species complex is difficult. Prior to genetic studies, the primary morphological method used to separate these two species was the DV/D ratio of male genitalia [15]. Molecular studies have since been developed such as rapid genetic assays which can be used to distinguish species within the complex [16–18], and the *ace*-2 gene can be useful to distinguish some populations more than others [16]. *Culex pipiens* and *Cx. quinquefasciatus* are known to hybridize. Hybrids of the two mosquito species might have biological traits of both parent species, which could broaden their host preference and increase the transmission and infection rates of West Nile virus [19, 20]. Hybrids have been challenging to identify as well. For example, neither the DV/D ratio nor the *ace*-2 gene could distinguish hybrid populations of the *Cx. pipiens* complex near Fresno, California [5].

Hybrids of *Cx. pipiens* and *Cx. quinquefasciatus* have been demonstrated to have higher transmission rates of WNv than those of each parent species [21]. For example, crosses of male × female *Cx. pipiens* had offspring with a 6% WNv transmission rate after a 13–14 d extrinsic incubation period (EIP), while progeny of male × female *Cx. quinquefasciatus* crosses had a 63.4% transmission rate [21]; hybrid offspring of female *Cx. quinquefasciatus* × male *Cx. pipiens* had even higher transmission rates (80.3%), while hybrids of female *Cx. pipiens* and male *Cx. quinquefasciatus* had a 62% transmission rate [21]. Similarly, hybrid offspring from *Cx. quinquefasciatus* or *Cx. pipiens* crossed with *Cx. pipiens molestus* had higher transmission rates than those from offspring of pure parental crosses. Hybrids of the *Cx. pipiens* complex are potentially a greater threat to public health if they have higher transmission rates of West Nile virus than offspring from pure parental crosses.

The proportion of hybrids and the WNv transmission rate have been investigated in the hybrid zone of *Cx. pipiens* and *Cx. quinquefasciatus* in the Central Valley of California. Using the DV/D ratio measurement of genitalia, mosquitoes identified as the *Cx. pipiens* complex from dairy lagoons in Merced County consisted of 11% *Cx. pipiens* complex hybrids and 89% *Cx. pipiens* [22]. Another study using the DV/D ratio to examine individuals from Merced found that 32% of the *Cx. pipiens* complex were classified as hybrids, 62% as *Cx. quinquefasciatus* and 6% were *Cx. pipiens* [3]. Variation in the percentage of *Cx. pipiens* to *Cx. quinquefasciatus* was found among multiple sites in Stockton, CA, suggesting population structure can occur on a relatively small scale [3]. Using field-collected mosquitoes from Fresno County and the *ace-*2 gene PCR assay for identification, 22% (98/442) of the *Cx. pipiens* complex collections were identified as hybrids; these field-collected hybrids had a 20% WNv infection rate (20/98 collected) compared with 21% (58/271) of those identified as *Cx. quinquefasciatus* or 14% (10/73) identified as *Cx. pipiens* [5]. However, the same study by McAbee et al. [5] used the DV/D ratio and found 66% (293/442) of individuals in Fresno classified as hybrids. In *Cx. pipiens* populations from Merced to Bakersfield, WNv transmission rates varied from 12.5 to 40% and 50 to 69% after an EIP of 7 and 14 d, while populations of ‘pure’ *Cx. pipiens* and *Cx. quinquefasciastus* from northern and southern California did not transmit West Nile virus [22]. Goddard et al. [1] found a Bakersfield population of *Cx. quinquefasciatus* had a higher transmission rate of WNv (52%) after 14 d EIP than did those from Riverside or Orange California (19 and 36%, respectively), while *Cx. pipiens* from Shasta had a 71% transmission rate after 14 d extrinsic incubation period [1]. The transmission rate of WNv for the *Cx. pipiens* complex varies among these studies. However, populations from the hybrid zone have typically demonstrated higher WNv transmission rates than pure parental populations, and transmission rates of WNv for *Cx. quinquefasciatus* are generally greater than for *Cx. pipiens* and *Cx. pipiens molestus* [1, 5, 21, 22].

Habitat can influence the species composition, the abundance of hybrids, and the WNv infection rate of the *Cx. pipiens* complex [23]. In other insect systems, adjoining habitats can contribute to genetically divergent populations, which has been termed ecological speciation [24, 25]. Similarly, mosquito populations with distinct host preferences can have genetic differences as well [26]. *Culex pipiens* and *Cx. pipiens molestus* can occur in different habitats in close proximity [13], demonstrating that habitat can be associated with the abundance of *Cx. pipiens* complex hybrids. *Culex pipiens molestus* is commonly associated with underground areas such as basements and sewers, yet recently hybrids of *Cx. pipiens* and *Cx. pipiens molestus* have been found indoors in animal shelters [8]. In California, previous studies have focused on hybridization of *Cx. quinquefasciatus* and *Cx. pipiens*; several studies have found evidence of *Cx. pipiens molestus* [12, 13]. The role of habitat in hybrid formation for *Cx. pipiens* and *Cx. quinquefasciatus* has rarely been investigated [27]. Although the proportion of hybrids in the *Cx. pipiens* complex was similar in urban and rural areas (34 and 36%, respectively), the abundance of the *Cx. pipiens* complex and the WNv infection rate was higher in urban low income areas than in urban middle income or in rural areas [23]. The general pattern of human WNv cases in North America has been that most cases occur in urban and agricultural (rural) habitats [28]. The Central Valley of California is largely rural, but habitats within Merced County vary greatly. Eastern Merced County has an urban corridor along a major highway and numerous dairies and potential hosts for *Cx. quinquefasciatus*, while western Merced County is rural with smaller communities, agricultural areas, and extensive wetland habitat for migrating birds, the preferred hosts of *Cx. pipiens*. Previous studies which included *Cx. pipiens* complex mosquitoes from Merced included few samples and varied greatly in the proportion of hybrids found. Given that laboratory studies have demonstrated that pure *Cx. quinquefasciatus* and its hybrids with *Cx. pipien*s have higher West Nile virus transmission rates, a more thorough investigation of the *Cx. pipiens* complex in the region would be beneficial.

The objective of this study was to determine the population genetic structure of *Cx. pipiens* complex mosquitoes in five habitats in Merced County, which is located in the Central Valley of California. This study investigated whether a particular habitat had a higher frequency of hybrids relative to abundance of pure *Cx. quinquefasciatus* or pure *Cx. pipiens*. Similarly, we were interested in whether any habitat had more pure *Cx. quinquefasciatus* due to its higher transmission rate of WNv than that of *Cx. pipiens*. Areas or habitats found to have more hybrids or more *Cx. quinquefasciatus* could be prioritized as targets for vector control.

## Methods

### Collecting samples

**Fig. 2:**
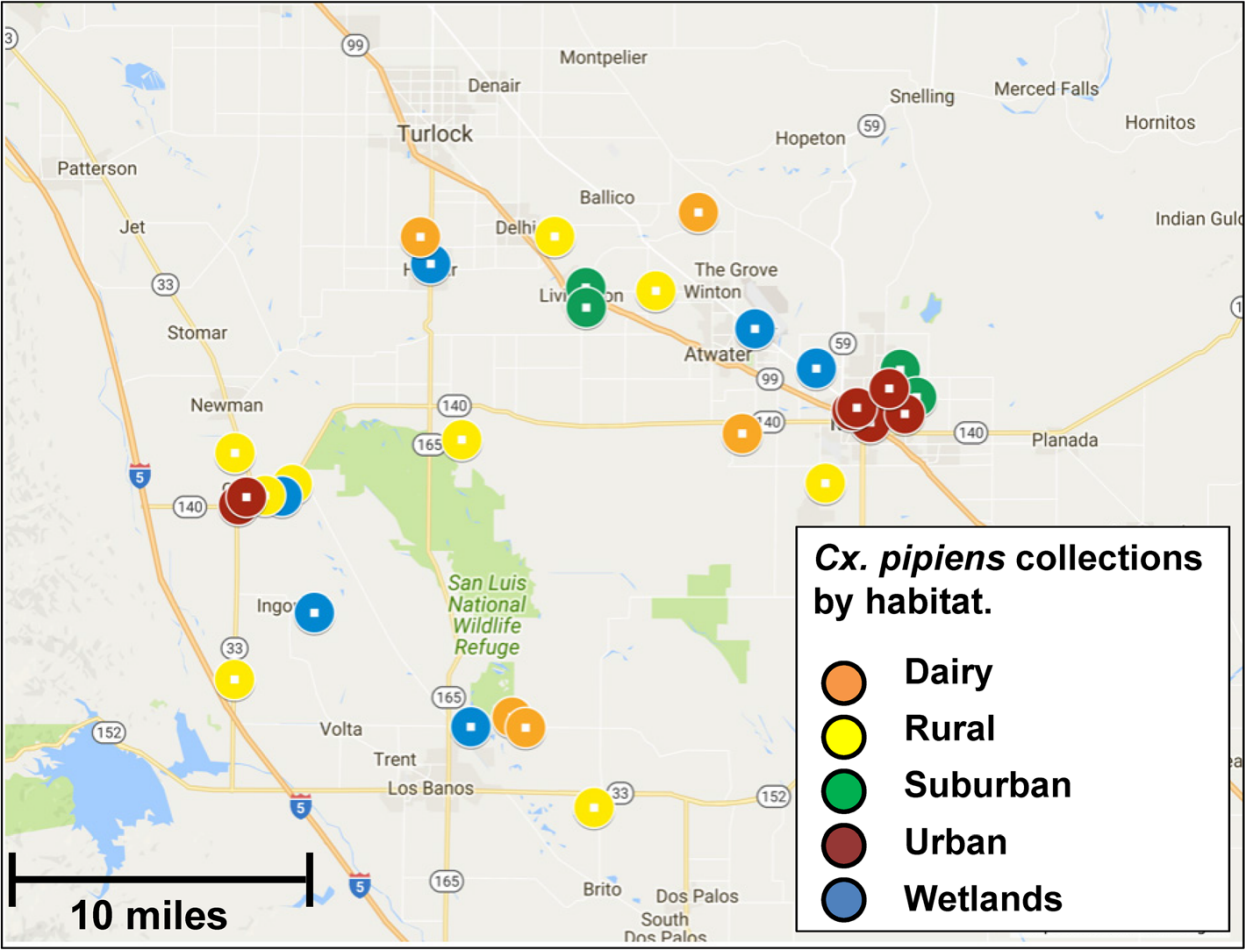
Collections of the *Cx. pipiens* complex in Merced County, California from five habitats. The habitats include dairies, rural, suburban, urban areas and wetlands

Mosquitoes from five habitats were collected in the hybrid zone of *Cx. pipiens* and *Cx. quinquefasciatus* in Merced County in the Central Valley of California (Figs. 1 and 2). Traps were set in four quadrants of the county; Northwest near Gustine, Southwest near Los Banos, Northeast near Hilmar and Livingston, and Southeast near Merced (Fig. 2). The distance across the sampled area in Merced County was 30 miles from North to South, and 30 miles from East to West. Mosquito samples were collected during 2012–2014. Each year, traps were set in the five habitats in each quadrant which included near dairies, rural, suburban, urban areas, and in wetlands. All traps were set in open outdoor areas with the intent to trap populations of *Cx. quinquefasciatus*, *Cx. pipiens*, and their hybrids; no traps were placed underground in sewers or basements, or in animal shelters, where *Cx. pipiens molestus* was presumed to occur. Dairies were typically in rural areas and had nearby dairy lagoons with runoff water polluted with manure. Rural areas were farmland or countryside with less than 2500 people in the area [29]. Suburban areas were residential areas located on the outskirts of a city or town [30], while urban areas were characterized by densely developed locations with at least 2500 residents [29]. Finally, wetlands were defined as land where water shallowly covers the soil at the surface and includes areas such as swamps and marshes [31]. Many collection site locations are sites regularly surveyed by the Merced County Mosquito Abatement District (MCMAD) surveillance program and are included in the California Vectorborne Disease Surveillance (CalSurv) database used for West Nile surveillance in California [32].

Adult female mosquitoes were trapped using CDC style light traps baited with CO_2_ set in the afternoon and retrieved the following morning during the months of June–October. Adult mosquitoes from traps were frozen and subsequently identified using light microscopy [33]. *Culex pipiens* and *Culex quinquefasciatus* are morphologically identical and thus classified as the *Culex pipiens* complex. The difficulty in distinguishing these two species morphologically, and the inconsistency of results using the DV/D ratio to separate hybrids of the two species, have led to a number of investigations using molecular markers to distinguish the two species. In Merced County, there are no other *Culex* species which resemble the *Culex pipiens* complex. Females identified as belonging to the *Cx. pipiens* complex were frozen or saved in ethanol for subsequent DNA extraction. Samples were collected from 31 unique sites in Merced County (Table 1), which were classified into five habitats of interest. The following number of collection sites within Merced county were used for each of the five habitats: dairy (*n* = 5), rural (*n* = 9), suburban (*n* = 5), urban (*n* = 7) and wetlands (*n* = 5) (Table 1, Fig. 2). Geographical coordinates of trap locations were recorded at each sampling site (Table 1, Fig. 2).

**Table 1:** Location of sample collections in Merced County by habitat

Habitat	Site name or site code CalSurv	Map code (Fig. 2)	GPS coordinates	Individuals used
Dairy	000402	D1	37.431944, -120.864167	2
	001225	D2	37.44205, -120.625528	42
	Baker Dairy	D3	37.105948, -120.781767	9
	F&A Brooks Dairy	D4	37.294767, -120.58755	11
	Stevenson/Hilmar	D5	37.0985, -120.7701933	8
	Total			72
Rural	001219	R1	37.426017, -120.745781	1
	000407	R2	37.255278, -120.992222	3
	000102	R3	37.261667, -120.518889	10
	001103	R4	37.389842, -120.661219	11
	Netherton	R5	37.2819444, -121.0138888	9
	000413	R6	37.045, -120.713056	2
	Old Romero School	R7	37.130233, -121.0147166	9
	Stevenson Ranch	R8	37.290767, -120.96598	8
	Gustine Airport	R9	37.260866, -120.96598	1
	Total			54
Suburban				
	909	S1	37.3375, -120.456111	6
	000506	S2	37.393333, -120.719444	16
	000507	S3	37.378611, -120.719444	4
	000403	S4	37.408056, -120.85	18
	KM’s House	S5	37.319008, -120.4422833	3
	Total			47
Urban	000916	U1	37.302222, -120.481389	8
	001205	U2	37.312183, -120.492539	11
	001227	U3	37.310103, -120.496792	4
	001304	U4	37.247411, -121.010942	1
	001301	U5	37.307947, -120.452553	6
	Sonora Pool	U6	37.324713, -120.465416	1
	Sycamore	U7	37.252366, -120.004333	4
	Total			35
Wetlands				
	000417	W1	37.338333, -120.526667	3
	001302	W2	37.099117, -120.816314	2
	000005	W3	37.364444, -120.578056	5
	Gustine Water Trtmnt	W4	37.252817, -120.973617	2
	Gustine Duck Club	W5	37.1750166, -120.947466	4
	Total			16

A population of *Cx. pipiens* from Washington State North of 39°, and one of *Cx. quinquefasciatus* from southern California South of 36° were included in the study for comparison with samples from our five Merced habitats. The pure *Cx. pipiens* population was collected from Lewis and Clark Reservoir, Washington (46°21′32″N, -119°25′29″E) and the *Cx. quinquefasciatus* population was collected from Mecca, Coachella, California (33°34′47″N, -116°4′37″E) (Fig. 1). We obtained a population from Oroville in northern California which we originally planned to use as the ‘pure’ *Cx. pipiens* population. However, the hybrid zone of *Cx. pipiens* and *Cx. quinquefasciatus* is now considered to extend North of its original 39° latitude boundary (and North of Oroville California), so we instead chose to use the Washington State population as our pure *Cx. pipiens* population (Fig. 1). All mosquito samples collected and used for DNA in this study were female.

### DNA extraction, amplified fragmented length polymorphisms (AFLPs)

DNA was extracted from the entire mosquito body (head, thorax, and abdomen) for all individuals using the Qiagen DNeasy Blood and Tissue kit (Venlo, Netherlands) following the protocols for animal tissue with an overnight incubation time of ~ 24 h at 65 °C [34]*.* Final products were eluted in 100 μl of AE buffer. The DNA quantity was measured using the Qubit® dsDNA HS Assay kit (Life Technologies-Thermo Fisher Scientific, Waltham, MA, USA). The quantity of DNA in samples averaged 5–10 ng/μl. Only female adults were used for molecular work.

Amplified fragment length polymorphisms were produced as described by Vos et al. [35] and modified by Joyce et al. [36]. Three primer combinations were used, (i) M-CAT and E-ACG, (ii) M-CAC and E-ACT and (iii) M-CAC and E-ACA (Table 2). Individuals from the five habitats (dairy, rural, suburban, urban and wetlands) in Merced, California and from three other locations [Mecca, Coachella, California (COA); Oroville California (ORO); and Lewis and Clark Reservoir, Washington (WA)] were all randomized on five 96-well plates for AFLP reactions. Eleven individuals were run in duplicate in order to test the error rate of the AFLP markers.

**Table 2:** Primer combinations used for selective polymerase chain reaction of amplified fragment length polymorphisms (AFLPs), number of markers produced by each primer combination, number < 125 bp, and the percent mismatch error rate

Primer combination	*EcoR*1-	*Mse*1-	No. of markers	Markers <125 bp	Percent mismatch
1	ACG	CAT	120	38/120	2.0
2	ACT	CAC	110	29/110	2.0
3	ACA	CAC	120	25/120	1.6

Each restriction/ligation reaction (well) consisted of the following: 0.05 μl each of *EcoR*I and *Mse*I, 1.1 μl of T4 DNA ligase buffer, 1.1 μl of 0.5 M NaCl, 0.55 μl of diluted BSA (bovine serum albumin), 0.03 μl of T4 DNA ligase, 1.0 μl each of *EcoR*I and *Mse*I adaptor pairs (Life Technologies-Thermo Fisher Scientific, Waltham, MA, USA), and 0.61 μl of sterile distilled water. The plate with restriction⁄ ligation reactions was held at room temperature overnight (12 h at 25 °C) to ensure complete digestion [37]. The amplified product was diluted 20-fold using 15 mM Tris-HCl buffer (pH 8.0) containing 0.1 mM EDTA. Pre-selective PCR amplification was performed on a ThermoFisher Arktik thermal cycler. Each reaction contained 15 μl of AFLP preselective mix (all Life Technologies/Thermo-Fisher), 1 μl of each amplification primer (Life Technologies), along with 4 μl of the diluted restriction⁄ ligation mixture. The PCR program for pre-selective amplification consisted of an initial warm-up of 95 °C for 1 min followed by 20 cycles at 95 °C for 20 s, 56 °C for 30 s, and 72 °C for 90 s with a final hold at 75 °C for 5 min. The amplified product was diluted 20-fold using 15 mM Tris-HCl buffer (pH 8.0) containing 0.1 mM EDTA. Selective amplification was conducted using two primer combinations. For each selective amplification, a reaction consisted of 15 μl of AFLP platinum supreme mix, 1.0 μl of *EcoR*I + 3 selective primers, and 1.0 μl of *Mse*I + 3 selective primers (all Life Technologies-Thermo Fisher Scientific Waltham, MA, USA)(Table 2). The PCR program for selective amplification consisted of an initial warm-up of 95 °C for 1 min, 12 cycles of 95 °C for 20 s, 65 °C for 40 s with a lowering of 0.7 °C per cycle, 72 °C for 90 s, followed by 35 cycles of 95 °C for 20 s, 56 °C for 40 s, 72 °C for 90 s, and finally a hold of 72 °C for 7 min before storing the samples at 4 °C. Prior to capillary electrophoresis, 0.4 μl of the Genescan LIZ 500 size standard and 0.9 μl of HiDi formamide (all Life Technologies) were added to 1 μl of the final product of each sample. The LIZ 500 size standard allows for detection of fragments between 50 and 500 bp. Sample fragments were separated using automated capillary electrophoresis by the ABI 3730 XL automated capillary DNA sequencer (Applied Biosystems-Thermo Fisher, Waltham, MA, USA).

GeneMapper version 5.0 (Life Technologies, ThermoFisher) was used to determine presence or absence of fragments. The peak detection threshold was set for each primer combination, and was typically 150 luminescent units. Each AFLP marker was considered a locus and assumed to have two possible alleles (0 = absent, 1 = present). Bands not present in more than one individual were eliminated (i.e. private alleles) prior to further analyses, as they were not considered informative. For samples which were run in duplicate, each marker was examined to determine whether markers were scored identically at each locus by GeneMapper, and data were used to calculate the mismatch error rate [38]. Structure 2.3.4 software [39] was used to group individuals with similar genotypes within each species. Structure uses a Bayesian algorithm to cluster individuals into K, which is defined as the number of genetically distinct populations in a data set. Parameters used for the analyses include the following: no a priori assignment of individuals to a known population, analysis for diploid insects, a burn-in of 100,000 and 200,000 subsequent iterations, an admixture model, and independent loci.

For runs in Structure software, the number of potential populations for K was estimated as the number of geographical sampling locations plus 4 as suggested by Pritchard et al. [40]. At the completion of Structure runs, K was calculated for each species using Structure Harvester using the Evanno method [41, 42], to determine the most likely number of population clusters (K) for the populations sampled. A Structure analysis was first run for all populations, and K was estimated as the number of geographic sampling locations plus 4 [5 Merced habitats + Oroville + pure *Cx. quinquefasciatus* + pure *Cx. pipiens* populations (8 pops +4, K = 12)] for the overall analysis as suggested by Pritchard et al. [40], and each iteration was run 20 times. A Structure analysis was also run for mosquitoes collected in each Merced habitat and compared to the *Cx. pipiens* and *Cx. quinquefasciatus* populations, as was the Oroville population. For each habitat analysis in Structure, the number of potential populations for K was estimated as the number of geographic sampling locations plus 4 (a single habitat + pure *Cx. quinquefasciatus* + pure *Cx. pipiens* populations = 3 populations +4, K = 7 for each habitat analysis) as suggested by Pritchard et al. [40]. Similarly, mosquitoes collected in all five Merced habitats were compared in an additional Structure analysis (K estimated as 5 habitats +4, K = 9), and examined with Structure Harvester as well. A q value of >0.80 from Structure was used to assign individuals to clusters while individuals with a q value <0.8 were considered admixed [13]. Structure results were used in Clumpak software to run Distruct to permutate runs to best visualize results.

New Hybrids v.1.1 software was used to examine the probability of each *Cx. pipiens* complex mosquito’s assignment of membership into a number of groups, including pure *Cx. quinquefasciatus*, pure *Cx. pipiens*, F1 hybrids of the two *Culex* species, F2 hybrids, or backcrosses to either parent [43]. Individuals were assigned to a pure species if q > 0.9, F1 if q = ~0.5, F2 if q < 0.5 for both parent species, and considered a hybrid backcross to a parent species if 0.5 ≤ q < 0.9. Individuals were not assigned a priori to a particular population, and runs were conducted with Jeffery like priors. One hundred thousand iterations were run and the posterior probability of each individual’s assignment to the above genetic classes was determined.

An analysis of molecular variation (AMOVA) was run to compare the molecular variation of individuals of the *Cx. pipiens* complex from populations collected in four regions, including *Cx. quinquefasciatus* (COA), *Cx. pipiens* (WA), Oroville California (ORO) and all individuals from Merced California [44]. The AMOVA was run using 999 permutations, and pairwise comparisons of the genetic divergence (F_ST_) values between populations were made, using Bonferroni corrections for multiple comparisons. A second AMOVA was run for individuals from the 5 Merced habitat populations also using 999 permutations. Similarly, the Fst values were compared for significance between pairs of populations, with Bonferroni corrections for multiple comparisons. Analyses were run using GenAlEx 6.5 software [45]. Output from the AMOVA Fst values were used to run a principal component analysis among the eight populations using GenAlEx 6.5.

Nei’s genetic distance was determined among all 8 populations using GenDist in Phylip 3.695 [46]. A Mantel test was run to determine if genetic distance was correlated to geographical distance between populations. Since collections from each Merced habitat came from 5 to 9 sites in the county (Table 1), we chose a representative location and genetic distance for Merced to run the Mantel test between Merced and the three other collection regions. We used the genetic distance from a downtown Merced urban population (U6) for comparison with Oroville CA, *Cx. pipiens* (WA) and *Cx. quinquefasciatus* (COA).

For *Cx. pipiens* complex mosquitoes from the five habitats, the proportion of unique alleles from *Cx. quinquefasciatus* (COA) and *Cx. pipiens* (WA) populations was determined for each Merced mosquito. Alleles unique to *Cx. quinquefasciatus* were defined as those which were present only in the pure *Cx. quinquefasciatus* population and not in in the pure *Cx. pipiens* population; similarly, loci unique to *Cx. pipiens* were those only found in the pure *Cx. pipiens* population. Unique loci from the pure *Cx. quinquefasciatus* and the pure *Cx. pipiens* population in each Merced mosquito were first identified. Next, the number of unique *Cx. quinquefasciatus* alleles and unique *Cx. pipiens* alleles were determined for individuals in each Merced habitat-associated population, and used to produce the mean proportion of *Cx. quinquefasciatus* and *Cx. pipiens* alleles for each of the five habitats and the Oroville population. For each habitat, we used a Chi-square goodness of fit test to compare whether the proportion of unique *Cx. pipiens*: *Cx. quinquefasciatus* alleles varied from a 1:1 equal distribution [47]. One-way ANOVA was then used to determine whether one habitat had a significantly higher proportion of *Cx. quinquefasciatus* alleles than other habitat-associated populations. Unique alleles which were found only in the Merced habitat populations but were not found in the Oroville, the pure *Cx. quinquefasciatus* or the *Cx. pipiens* populations were also identified as well. Loci present in higher frequency and found in nearly half the individuals (30 or more) of at least 2 or more habitat-associated populations were determined, followed by unique alleles found in very low frequencies (typically in 1–10 individuals) in the Merced populations.

The software Mcheza was used to examine candidate loci that may be under selection in the habitat associated populations [48]. Mcheza is available from popgen.net, and is a selection workbench developed for dominant markers such as AFLPs. The file with presence or absence of AFLP loci was converted to the Genepop format. MCheza was run to examine which loci were Fst outliers. The following default settings were used; confidence interval 0.95, false discovery rate of 0.1, Theta 0.1, beta-a 0.25 and beta-b 0.25. The option of neutral mean Fst was chosen and 500,000 simulations were run. Candidate loci under positive selection, balancing selection and neutral selection were determined.

## Results

### Collecting samples

Female mosquitoes identified as the *Culex pipiens* complex were collected from 5 dairies, 9 rural, 5 suburban, 7 urban and 5 wetland sites in Merced County (Table 1, Fig. 2). *Culex pipiens* complex mosquitoes were generally more abundant from collections near dairies and were relatively common in rural, suburban and urban samples (Table 1). Most wetlands collections in this study yielded few *Cx. pipiens* complex mosquitoes, as the wetlands sites were dominated by *Culex tarsalis* Coquillett.

### Population genetic structure: amplified fragment length polymorphisms (AFLPs)

Amplified fragment length polymorphisms (AFLPs) were produced using 3 primer combinations (Table 2). There were 350 alleles produced for comparison of the 278 individuals from the 8 populations in the study, which included Coachella California (*Cx. quinquefasciatus*), 5 habitat-associated populations of *Cx. pipiens* complex from Merced CA, Oroville CA (*Cx. pipiens* complex) and *Cx. pipiens* from Washington State. From Merced County, there were 72 *Cx. pipiens* complex mosquitoes included from dairies, 54 from rural areas, 52 from suburban areas, 35 from urban sites and 16 from wetland habitats. There were also 17 mosquitoes of pure *Cx. quinquefasciatus* from Coachella, 17 *Cx. pipiens* complex from Oroville, and 15 pure *Cx. pipiens* from Washington. Fragments produced were viewed with GeneMapper 5.0 and scored as present or absent for each individual. Three primer combinations were used to produce AFLPs, resulting in 120, 110, and 120 fragments (Table 2). The number of fragments which were sized <125 bp for each primer were 38/120 for primer combination 1, 29/110 for primer 2, and 25/120 for primer 3 (Table 2). A test of the error mismatch rate for the AFLP markers for 8 individuals from Merced found that fragments had a 2, 2 and 1.6% mismatch error rate for each of the three primers, respectively (Table 2).

**Fig. 3:**
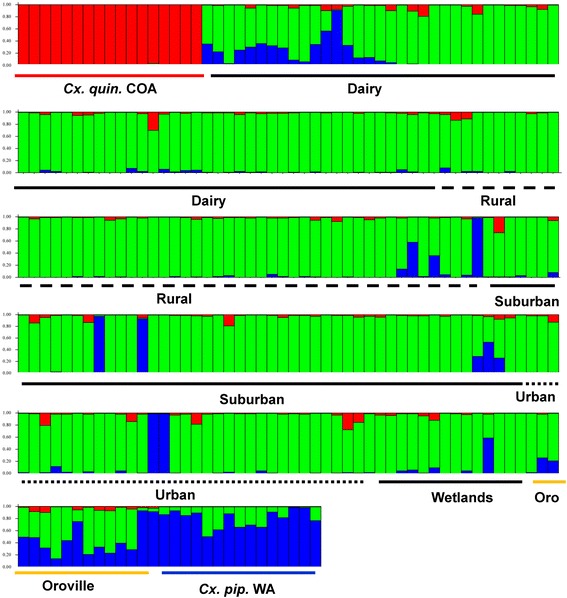
A Structure 2.3.4 analysis of the 8 populations in the study was run using the following parameters: diploid individuals, 100,000 iterations, admixed data, and independent loci. Each vertical bar represents an individual mosquito. The y-axis shows the probability of an individual being assigned to one of the three genetic clusters. Red bars (*Cx. quin*) represent the *Cx. quinquefasciatus* individuals from Coachella, California. Green bars represent individuals from five habitat-associated populations from Merced; dairy, rural, suburban, urban and wetlands. Oroville (Oro) California is a mixture of green and blue bars, and blue bars (*Cx. pip*) represent individuals of *Cx. pipiens* from Washington. Structure Harvester found that K = 3; there were three genetically distinct populations

**Fig. 4:**
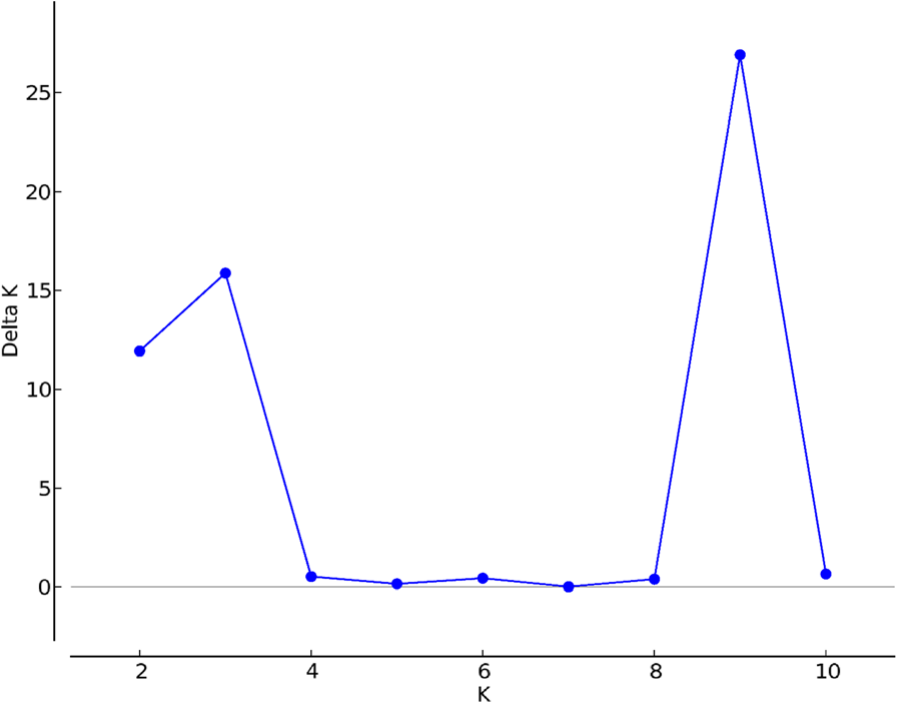
Results from a Structure Harvester analysis of all eight populations. Structure Harvester uses the results from Structure to calculate the Delta K value, the change in likelihood, for the number of potential clusters. Structure Harvester calculated the most likely number of clusters was 3 (K = 3)

The Structure analysis of all 8 populations and subsequent Structure Harvester analysis of the data found the highest Delta K Evanno value at K = 9, followed by K = 3 (Figs. 3 and 4, Additional file 1: Figure S1). However, the result of K = 9 populations was not biologically relevant, so the value of K = 3 was chosen indicating that there were 3 genetically distinct groups (Figs. 3 and 4, Additional file 1: Figure S1, Additional file 2: Figure S2). *Culex quinquefasciatus* from Coachella California formed the first group, the Merced and Oroville California collections formed a second central valley group, and the last cluster was *Cx. pipiens* from Washington (Fig. 3). The Structure analysis found that all individuals of *Cx. quinquefasciatus* from Coachella California had a q value of >0.8 and were considered pure *Cx. quinquefasciatus* (Fig. 3), and most individuals of *Cx. pipiens* from Washington had q values >0.8. From the Washington *Cx. pipiens* population, there were 6 individuals considered admixed. Nearly all individuals from Merced classified their own cluster (97%, 233/237 green) (Fig. 3). The *Cx. pipiens* complex mosquitoes from the Merced habitats had 3% (6/239) individuals that classified as pure *Cx. pipiens* (blue bars); one was from dairy, one from rural, two from suburban and two from urban areas (Fig. 3). None of the mosquitoes from Merced habitats classified as pure *Cx. quinquefasciatus*. Oroville CA mosquitoes all classified as admixed (Fig. 3).

Subsequent Structure analyses compared individuals from each Merced habitat to those of the pure *Cx. pipiens* and *Cx. quinquefasciatus* populations. The Structure Harvester analysis for each habitat found the number of genetically distinct populations was K = 2; individuals from each Merced habitat shared alleles with both *Cx. pipiens* and *Cx. quinquefasciatus,* suggesting hybrid populations in each Merced habitat as well as in Oroville CA (Additional file 3: Figure S3a-f). In all habitat analyses and the Oroville analysis, the q values were >0.9 for all *Cx. quinquefasciatus* from Coachella California, and >0.9 for all *Cx. pipiens* from Washington. In the Structure analysis of mosquitoes from dairy habitat, most (69%, 50/72) were admixed, while 31% (22/72) had q values >0.9 for assignment to pure *Cx. pipiens* (Additional file 3: Figure S3a). For rural areas, most (88%, 45/51) were admixed, and 12% (6/51) of mosquitoes had q values >0.9 for assignment to *Cx. pipiens* (Additional file 3: Figure S3b). For suburban mosquito collections, most 85% (40/47) again were admixed, and 15% (7/47) of mosquitoes were assigned to *Cx. pipiens* (Additional file 3: Figure S3c). Urban mosquitoes followed a similar pattern; 89% of urban mosquitoes (31/35) were admixed, and 11% (4/35) classified as *Cx. pipiens* (Additional file 3: Figure S3d). Finally, wetlands had 100% of individuals admixed (16/16) (Additional file 3: Figure S3e). The collection from Oroville California had 65% (11/17) of individuals admixed, and 35% (6/17) classified as *Cx. pipiens* (Additional file 3: Figure S3f). An additional Structure and Structure Harvester analysis of the five Merced habitat-associated populations found the highest Evanno Delta K value was at K = 8, followed by K = 6 and K = 4; however, all Structure graphic files illustrating the probability of assignment for individuals for all of the aforementioned K values indicated one genetic cluster, and it was concluded that K = 1.

The software New Hybrids was used to classify the ancestry of mosquitoes into groups, and determine whether they were parental species, F1 or F2 hybrids, or backcrosses to pure parental species. The analysis found the Coachella Ca mosquitoes were all classified as pure *Cx. quinquefasiatus* (100%), and the Washington mosquitoes were all classified as parental *Cx. pipiens*, as expected (Table 3). Individuals from Merced dairies had 3% (2/72) assigned to pure *Cx. pipiens,* 0% assigned to *Cx. quinquefasciatus*, 0% F1 or F2 hybrids, and 97% (70/72) assigned to hybrid backcrosses to pure *Cx. pipiens*. Rural areas in Merced had 2% (1/51) assigned to *Cx. pipiens*, 0% to *Cx. quinquefasciatus*, 0% F1 and F2, and 98% (50/51) were hybrids backcrossed to *Cx. pipiens*. Suburban areas in Merced had 100% (47/47) classify as hybrids backcrossed to *Cx. pipiens*, as did those from urban areas (35/35) and wetlands mosquitoes (16/16). Finally, Oroville had all but one (16/17, 94%) individual classified as hybrids backcrossed to *Cx. pipiens*.

**Table 3:** Frequency of individuals in pure and hybrid classes from a New Hybrids analysis

	Pure-bred A *Cx. pipiens*	Hybrids				Pure-bred B *Cx.quinque*
Lineage	Pure A	F1	F2	Backcross A	Backcross B	Pure B
*Cx. pipiens*	15/15	0	0	0	0	0
Dairy	2/72	0	0	70/72	0	0
Rural	1/51	0	0	50/51	0	0
Suburban	0	0	0	47/47	0	0
Urban	0	0	0	35/35	0	0
Wetland	0	0	0	16/16	0	0
Oroville	1/17	0	0	16/17	0	0
*Cx. quinquefasciatus*	0	0	0	0	0	17/17

### Analysis of molecular variation (AMOVA)

AMOVA of the *Cx. pipiens* complex populations from four geographic areas found that collection region had a significant effect on genetic variation (Table 4), accounting for 12% of variation. Pairwise Fst values of genetic divergence between the four groups were significant (*P* < 0.01). The genetic distance estimate between *Cx. quinquefasciatus* from Coachella and *Cx. pipiens* from Washington was 0.360, *Cx. quinquefasciatus* from Coachella to Merced and Oroville were 0.164 and 0.232, respectively, and *Cx. pipiens* from Washington to Oroville and Merced was 0.108 and 0.106, respectively (Table 5). Finally, Merced and Oroville which are both considered to be in the hybrid zone were less genetically distant (0.046) from each other than with the other populations.

**Table 4:** Results of analysis of molecular variation (AMOVA) tests

Source	*df*	Sum of squares	Variation (%)	*P*
Among regions^a^	3	563.80	12	0.001
Individuals within regions	274	10,694.66	88	
Among habitats^b^	4	204.86	1	0.008
Individuals within habitats	224	8904.05	99	

^a^*Cx. pipiens* complex populations from four regions including *Cx. quinquiefasciatus* from Coachella CA, *Cx. pipiens* complex from Merced and Oroville, and *Cx. pipiens* from Washington

^b^Populations of *Cx. pipiens* complex from five Merced habitats

*Abbreviation*: *df* degrees of freedom

**Table 5:** Results of pairwise comparisons of genetic divergence estimates (F_ST_) between *Cx. pipiens* complex populations

	Population	1	2	3	4
1	*Cx. quinquefasciatus* COA	0			
2	*Cx. pipiens* complex Merced	0.164*	0		
3	*Cx. pipiens* complex ORO	0.232*	0.046*	0	
4	*Cx. pipiens* WA	0.360*	0.106*	0.108*	0

^*^P < 0.01 indicates comparison between populations is significant. All values were significant at *P* < 0.01 after a Bonferroni correction

*Abbreviations*: *COA* Coachella CA, *ORO* Oroville CA, *WA* Lewis and Clarke Reservoir, WA

Molecular variation between the five Merced habitat collections was significant as well (AMOVA, *P* = 0.008), and accounted for 1% of the variation (Table 4). Pairwise genetic divergence tests found that individuals collected near dairies were significantly different than those collected near suburban and urban areas (*P* < 0.01) (Table 6). Pairwise comparisons among genetic divergence of other habitats were not significantly different.

**Table 6:** Results of pairwise comparisons of genetic divergence estimates (F_ST_) from five Merced habitats

	Populations in Merced habitat	1	2	3	4	5
1	Dairy	0				
2	Rural	0.004 ns	0			
3	Suburban	0.007*	0.001 ns	0		
4	Urban	0.011*	0.008 ns	0.006 ns	0	
5	Wetland	0.009 ns	0.011 ns	0.010 ns	0.008 ns	0

^*^P < 0.01 indicates comparison between populations is significant. Results were corrected for multiple comparisons with a Bonferroni correction

*Abbreviation*: *ns* not significant at *P* < 0.01

**Fig. 5:**
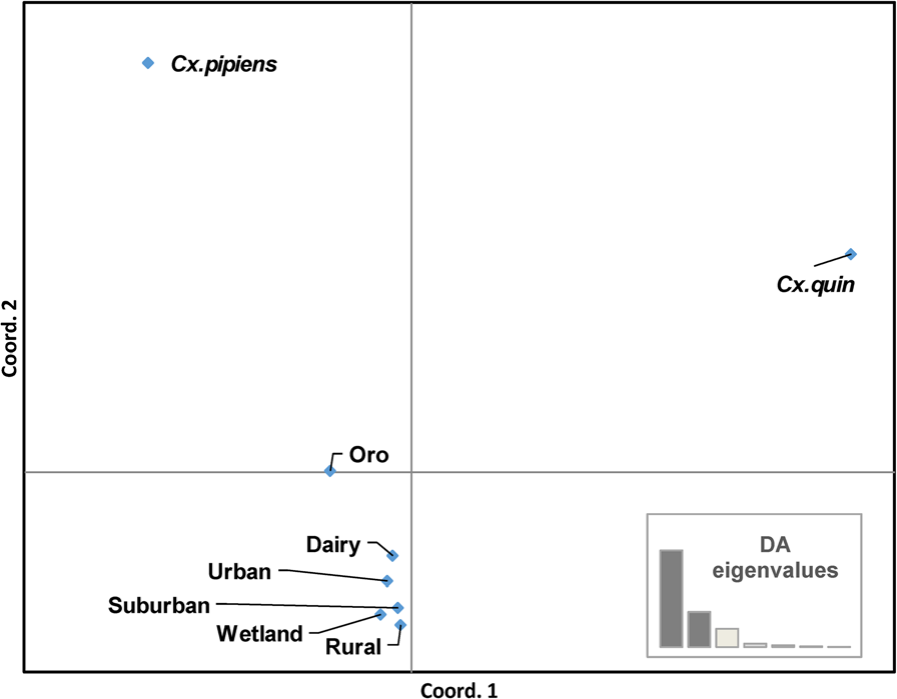
Principal components analysis using genetic distance output of AMOVA of the eight populations in the study. The eight populations included pure *Cx. pipiens* from Washington, pure *Cx. quinquefasciatus* (*Cx. quin*) from Coachella, California, a population from Oroville (Oro) Ca, and five habitat-associated populations from Merced

The principal components analysis found that the first axis accounted for 61.82% of the variation, while the second and third axes explained 22.56% and 11.77% of the variation, respectively (Fig. 5). Examining axis 1, from left to right, there is a clear separation of the *Cx. pipiens* and *Cx. quinquefasciatus* populations into the top two quadrants (Fig. 5). The population from Oroville nearly clustered into the quadrant with *Cx. pipiens*, and it was intermediate between the *Cx. pipiens* population and the Merced habitat-associated populations. The second axis (y-axis) separates the *Cx. pipiens* population from the Merced habitat associated populations, which fall into a third quadrant, yet are positioned on axis 1 between *Cx. pipiens* and *Cx. quinquefasciatus* (Fig. 5).

### Genetic distance and mantel test

Nei’s genetic distance was determined between all 8 populations in the study (Table 7). The largest genetic distance was between the *Cx. pipiens* and *Cx. quinquefasciatus* populations from Coachella California and Washington (0.134, 13%). *Culex quinquefasciatus* from southern California was 0.064–0.067 distant from the five habitats in Merced and 0.087 distant from Oroville. *Culex pipiens* from Washington was 0.05 distant from Oroville, California and 0.042–0.49 distant from Merced populations. Finally, the Merced populations were 0.021–0.032 distant from Oroville (Table 7). A Mantel test found no significant relationship between the genetic distance and geographic distance for the *Cx. pipiens* populations (*r* = 0.686, *P* = 0.110).

**Table 7:** Nei’s genetic distance among populations. Populations from Coachella, California (COA), Merced County populations from dairy, rural, suburban (Suburb.), urban and wetland (Wet.) areas, Oroville California, and Lewis and Clark Reservoir, Washington (L&C, WA). Locations of all populations in Table 1 and Fig. 2

	COA	Dairy	Rural	Suburb.	Urban	Wet.	Oroville	L&C,WA
COA.	–	0.064	0.064	0.065	0.067	0.067	0.087	0.134
Dairy		–	0.006	0.007	0.009	0.012	0.021	0.042
Rural			–	0.006	0.009	0.014	0.027	0.048
Suburb				–	0.009	0.014	0.028	0.049
Urban					–	0.014	0.032	0.045
Wetland						–	0.030	0.048
Oroville							–	0.050
L&C, WA								–

### Proportion of unique *Cx. quinquefasciatus* and *Cx. pipiens* alleles by habitat

The proportion of alleles unique to the COA *Cx. quinquefasciatus* and WA *Cx. pipiens* populations were determined for each individual from the five Merced habitats (Table 8). The number of unique alleles in the pure *Cx. quinquefasciatus* population which were not found in pure *Cx. pipiens* was 73, while 122 unique alleles were found in the pure *Cx. pipiens* which were not present in the pure *Cx. quinquefasciatus* population. For each of the five habitat-associated populations in Merced, the average number of *Cx. quinquefasciatus* alleles in all individuals in each populations was 9, while the average number of *Cx. pipiens* alleles was 15; Oroville individuals had an average of 10 unique alleles from *Cx. quinquefasciatus* and 20 from *Cx. pipiens*. The proportion of unique *Cx. quinquefasciatus* alleles in Merced populations ranged from an average of 38% in dairy and urban populations up to 42% in rural populations (Table 8), while the proportion of unique *Cx. pipiens* alleles ranged from 0.58 in rural areas to 0.62 in dairies (Table 8). Within each habitat, a Chi-square test found that the proportion of *Cx. pipiens: Cx. quinquefasciatus* varied significantly from 1:1 (*P* < 0.05) (Table 8), with a lower proportion of alleles contributed from *Cx. quinquefasciatus* than from *Cx. pipiens*. A one-way ANOVA found there was no significant difference in the proportion of unique *Cx. quinquefasciatus* alleles among all 5 habitat-associated populations (*F*_(4,265)_ = 0.65, *P* = 0.623). The Oroville population had a larger proportion of *Cx. pipiens* alleles than of *Cx. quinquefasciatus* (*χ*^2^ = 38.72; *df* = 1; *P* < 0.001) (*Cx. pipiens* 72%, *Cx. quinquefasciatus* 28%) (Table 8).

**Table 8:** The proportion of fixed unique alleles derived from pure populations of *Cx. pipiens* and *Cx. quinquefasciatus*

Habitat	No. of individuals	Proportion of *Cx. pipiens* alleles	Proportion of *Cx. quinquefasciatus* alleles	*χ*^2^-value	*P*-value
Dairy	72	0.620	0.380	11.49	0.0007*
Rural	54	0.580	0.420	5.12	0.024*
Suburban	47	0.600	0.400	8.00	0.005*
Urban	35	0.620	0.380	11.49	0.0007*
Wetlands	16	0.625	0.385	10.58	0.001*

^*^P < 0.05

**Fig. 6:**
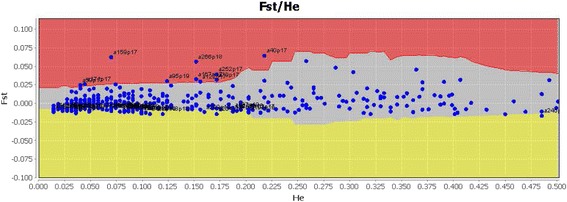
Loci under possible positive selection shown in red, neutral selection in the gray region, and those under balancing selection are shown in the yellow region, using Mcheza software

Merced habitat-associated populations and the Oroville population were examined as well to determine their unique alleles. Merced habitats had three alleles at relatively high frequency, which were found in nearly half the individuals in 2 or 3 habitats, yet they were not present in the Oroville population, nor in the *Cx. pipiens* or *Cx. quinquefasciatus* populations. One of these loci was present in 34, 30, 30, 15 and 9 individuals of dairy (D), rural (R), suburban (S), urban (U) and wetland (W) habitat collections, respectively, while a second and third unique loci were found in 31D, 21R, 3S, 4 U, 7 W and 1D, 16R, 13S, 6 U and 0 W individuals of the same habitats. In additional, there were fifteen alleles found at a very low frequency (in 1–10 individuals) in several habitats in Merced, and which were also not found in the Oroville, *Cx. pipiens*, or the *Cx. quinquefasciatus* populations. The Mcheza analysis to examine loci under selection found 10 loci under putative positive selection, and 36 loci possibly under balancing selection (Fig. 6).

## Discussion

The *Cx. pipiens* species complex consists of morphologically similar, but genetically, behaviorally and ecologically distinct populations. Numerous studies have considered whether *Cx. pipiens* and *Cx. quinquefasciatus* are distinct species or subspecies of *Cx. pipiens*, especially with respect to variation in host feeding preference and reproductive diapause [49, 50]. Previous studies used the DV/D ratio of the genitalia to separate these two groups and their hybrids [15], but environmental conditions and food availability could influence the size of individuals in a population, and subsequent work found that DV/D ratios do not consistently correspond to genetic differences in populations [3, 5]**.** In the case of a species complex, molecular markers are helpful and could be more accurate to separate populations within a species group. Different insect orders vary in the level of genetic variation considered sufficient to warrant separate species status [51, 52]. A study of *Culex annulirostris* in Australia and Papua New Guinea found morphologically similar but genetically divergent lineages just 3% divergent, one able to transmit Japanese encephalitis virus (JEV) while another lineage did not [53]. In this study, *Cx. pipiens* and *Cx. quinquefasciatus* had a genetic distance of 0.134 (13%) indicating moderate genetic divergence, which supports that these two groups are distinct species [49].

The Structure analysis of all eight populations considered in this study found K = 3 (three distinct groups) with Merced and Oroville populations in the Central Valley more similar to each other than to either the *Cx. pipiens* or *Cx. quinquefasciatus* populations. The AMOVA analysis found 12% of molecular variation among the eight populations in this study, similar to the 11% and 10% variation observed in two previous studies of *Cx. pipiens* populations in the Midwest and in California, USA [13, 20]. The populations in this study from the *Cx. pipiens* complex hybrid zone had genetic distances between *Cx. pipiens* and *Cx. quinquefasciatus* in the range of 0.04–0.05, close to 5%, suggesting the stable interbreeding hybrid population is undergoing significant divergence from *Cx. pipiens* and *Cx. quinquefasciatus*. This is also supported by the finding of a number of loci putatively under positive selection (Fig. 6). Hybrid offspring of other species are often sterile, but the *Cx. pipiens* complex hybrids in the Central Valley are fertile and can interbreed with both *Cx. pipiens* and *Cx. quinquefasciatus* [13]. An interesting biological attribute of *Cx. pipiens* and *Cx. quinquefasciatus* is their ability to hybridize in areas where they have been introduced, yet not to hybridize in South Africa where they are thought to have originated, and where they occur together sympatrically [4].

A stable interbreeding population in the hybrid zone of California has been observed previously [13]. A study of the population genetic structure of the *Cx. pipiens* complex from southern to northern California and also including Washington found four genetically distinct groups, with two clusters in the Central Valley. One Central Valley California population which occurred in collections from northern California near Shasta and South to Turlock was called Cluster X, which Merced *Cx. pipiens* hybrids may belong to. The F_ST_ pairwise genetic divergence estimates in this study for *Cx. quinquefasciatus* from Coachella and *Cx. pipiens* from Oroville were 0.236, while those of Kothera et al. [13] for *Cx. quinquefasciatus* from Coachella and *Cx. pipiens* from Shasta were 0.27. From the Coachella population to that of Merced, the pairwise genetic divergence estimate was 0.164, while in Kothera et al. [13] their measurements for two Coachella populations to Turlock ranged from 0.174 to 0.187. The study by Kothera et al. [13] used microsatellites, while this study determined genetic structure using AFLPs. The similarity of results between the two studies supports the utility of both types of molecular markers for population comparisons.

We sampled different ecological habitats of Merced to determine whether *Cx. pipiens* and *Cx. quinquefasciatus* could occur in close proximity in different habitats, since they were found to occur together in previous studies of the Central Valley of California, as well as in South Africa [3–5, 22]. In Merced, the majority of individuals sampled were hybrid mosquitoes; however, dairy populations had more pure *Cx. pipiens* individuals. Populations in all five habitats had more alleles specific to the *Cx. pipiens* population from Washington than alleles unique to *Cx. quinquefasciatus* from Coachella, even though the geographical distance between Merced and Washington is almost twice the distance as between Merced and Coachella, California (~700 miles *vs* ~400 miles). Another study of *Cx. pipiens* through the middle of the USA similarly found that in Memphis, near the center of the hybrid zone, nearly all individuals sampled were hybrids [20].

Habitats where *Cx. pipiens* complex mosquitoes were collected in Merced had a small but significant influence on the genetic composition of hybrids. AMOVA analysis revealed 1% of genetic variation in Merced collections due to habitats, with dairy collections being genetically divergent from rural and suburban habitats. The analysis which examined the number of unique *Cx. pipiens* or *Cx. quinquefasciatus* alleles in the Merced populations found that on average each habitat had a similar proportion of *Cx. pipiens*: *Cx. quinquefasciatus* alleles, with rural collections having a trend toward a higher percentage of *Cx. quinquefasciatus* alleles (42%); perhaps this small but statistically insignificant difference is enough to influence where human WNv cases occur.

There were several alleles found in the Merced populations at relatively high frequency, and fifteen alleles found in the Merced populations at low frequency, none of which were found in the *Cx. pipiens*, *Cx. quinquefasciatus* or the Oroville populations. The principal components analysis found that *Cx. pipiens* and *Cx. quinquefasciatus* fell into separate quadrants. However, the Merced habitat associated populations were in a third quadrant, but with respect to the x-axis, they were in close proximity to being intermediate between *Cx. pipiens* and *Cx. quinquefasciatus*. There are several explanations as to why the Merced hybrids are not placed exactly in between the two upper quadrants with *Cx. pipiens* and *Cx. quinquefasciatus*. The Merced populations consist of many more individuals than included in the *Cx. pipiens* or *Cx. quinquefasciatus* populations, which might have captured some alleles not present in the smaller parent populations. However, the Oroville population was similar sized to the *Cx. pipiens* and *Cx. quinquefasciatus* populations, and it lies between the *Cx. pipiens* quadrant and the third quadrant with the Merced populations. It is unlikely that the Merced mosquito populations are misidentified; there are no known mosquito species in the Merced area which could be confused morphologically with the *Cx. pipiens* complex. The placement of the Merced populations in the third quadrant in the principal component analysis suggests a genetic contribution from a closely related member of the *Cx. pipiens* complex, perhaps *Cx. pipiens molestus*.

Previous studies of the *Cx. pipiens complex* in the Merced area did not consider the presence of *Cx. pipiens molestus*. We focused this study on whether *Cx. quinquefasciatus* or *Cx. pipiens* complex hybrids might be more common in a particular habitat; for that reason, mosquito collections were focused outdoors in aboveground habitats where it was believed that *Cx. pipiens molestus* would not commonly occur. However, several recent studies including have found *Cx. pipiens molestus* hybrids more widespread and in more habitats than previously expected [8, 13]. In this study, Merced collections did not include sewers or underground structures such as basements which *Cx. pipiens molestus* was traditionally considered to inhabit. Future studies in Merced and the southern San Joaquin Valley should collect underground in sewers and basements along with other outdoor habitats including in animal shelters, to determine where *Cx. pipiens molestus* may be present.

The genetic composition of hybrids is just one factor that could influence where WNv positive mosquitoes are abundant. Most hybrids were of similar genetic composition, consisting primarily of hybrids backcrossed to *Cx. pipiens*. Thus, they may be likely to have similar vector capacities and transmission rates of West Nile virus. However, this would need to be determined experimentally to ascertain with certainly the ability of hybrids backcrossed to parental species to transmit West Nile virus.

Temperature has been suggested as a limiting environmental factor which affects the distribution of *Cx. pipiens* and *Cx. quinquefasciatus* along with the extent of their hybrid zone [5]. Warmer winters in southern latitudes allow *Cx. quinquefasciatus* to overwinter as reproductive adults, which contributes to higher *Cx. quinquefasciatus* abundance in the spring, more generations per year, and a longer WNv disease transmission season. In the Central Valley of California, cooler winter temperatures may prevent *Cx. quinquefasciatus* from surviving. In northern latitudes where *Cx. pipiens* is abundant, adults undergo reproductive diapause and are less numerous in spring. In addition, *Cx. pipiens* have a low vertical transmission rate of West Nile virus, making the disease slower to increase each year in local mosquito populations [5]. Some have suggested the southern range of the hybrid zone has moved North of 39° latitude [20]. The Oroville population in this study which was collected near 39° latitude was found to be a hybrid population, not pure *Cx. pipiens*. Models of climate warming and increasing average temperatures predict the northern expansion of *Cx. pipiens* complex hybrids, and an increasing number of human West Nile virus cases [53]. Studies have demonstrated variation in vector capacity of *Cx. pipiens* complex populations, with hybrids having higher transmission rates than non-hybrids, and pure *Cx. quinquefasciatus* having among the highest transmission rates [5, 21].

## Conclusions

This study investigated the hybrid composition of *Cx. pipiens* complex populations from five Merced habitats to determine whether some habitats had more hybrids than others. Nearly all mosquitoes collected were hybrids backcrossed to *Cx. pipiens*; collections from dairies had more individuals than the other habitats that classified as pure *Cx. pipiens*, while none of the habitats in Merced or Oroville had mosquitoes that classified as pure *Cx. quinquefasciatus*. Mosquitoes collected in dairies were genetically divergent from those collected in suburban and urban areas. All hybrids had a larger proportion of alleles shared with the pure *Cx. pipiens* population than with the pure *Cx. quinquefasciatus* population. Results also suggest the presence of *Cx. pipiens molestus* in Merced, but this needs to be confirmed. The *Cx. pipiens* complex in the Merced shared more alleles with the pure *Cx. pipiens* population, even though it was more geographically distant from the pure *Cx. pipiens* populations than from the pure *Cx. quinquefasciatus* population. Cold winter temperatures may limit the northern introgression of *Cx. quinquefasciatus* alleles into the hybrid zone. Future studies might examine the ability of hybrids backcrossed to *Cx. pipiens* and *Cx. quinquefascitus* to transmit West Nile virus.

## Additional files

Additional file 1: Figure S1. Results from a Structure Harvester analysis of all eight populations of the *Cx. pipiens* complex in this study. Each row shows the probability of K populations and delta K. The most likely number of populations was K = 3. (TIFF 2681 kb)

Additional file 2: Figure S2. The results from Distruct using output from Structure for K = 3 populations. (TIFF 2317 kb)

Additional file 3: Figure S3. Structure analyses for each of five Merced habitat-associated populations and for the Oroville California population individually compared to *Cx. quinquefasciatus* from Coachella, California and to *Cx. pipiens* from Washington. Structure was run using the following parameters: diploid individuals, 100,000 iterations, admixed data, and independent loci. Each vertical bar represents an individual mosquito. Structure Harvester found K = 2 clusters. The y-axis shows the probability of an individual being assigned to one of the two genetic clusters. Panels include **a** dairy (D) collections, **b** rural (R) habitat collections, **c** suburban (S) collections, **d** urban (U) collections, **e** wetland (W) collections, and **f** Oroville California, each compared to pure *Cx. quinquefasciatus* and *Cx. pipiens* populations. (PDF 659 kb)
