# Supplementary figures and images for: Population genetic structure of the Culex pipiens (Diptera: Culicidae) complex, vectors of West Nile virus, in five habitats
Source: Parasit Vectors. 2018 Jan 4;11:10. doi: 10.1186/s13071-017-2594-6 (PMC5755309; doi:10.1186/s13071-017-2594-6)

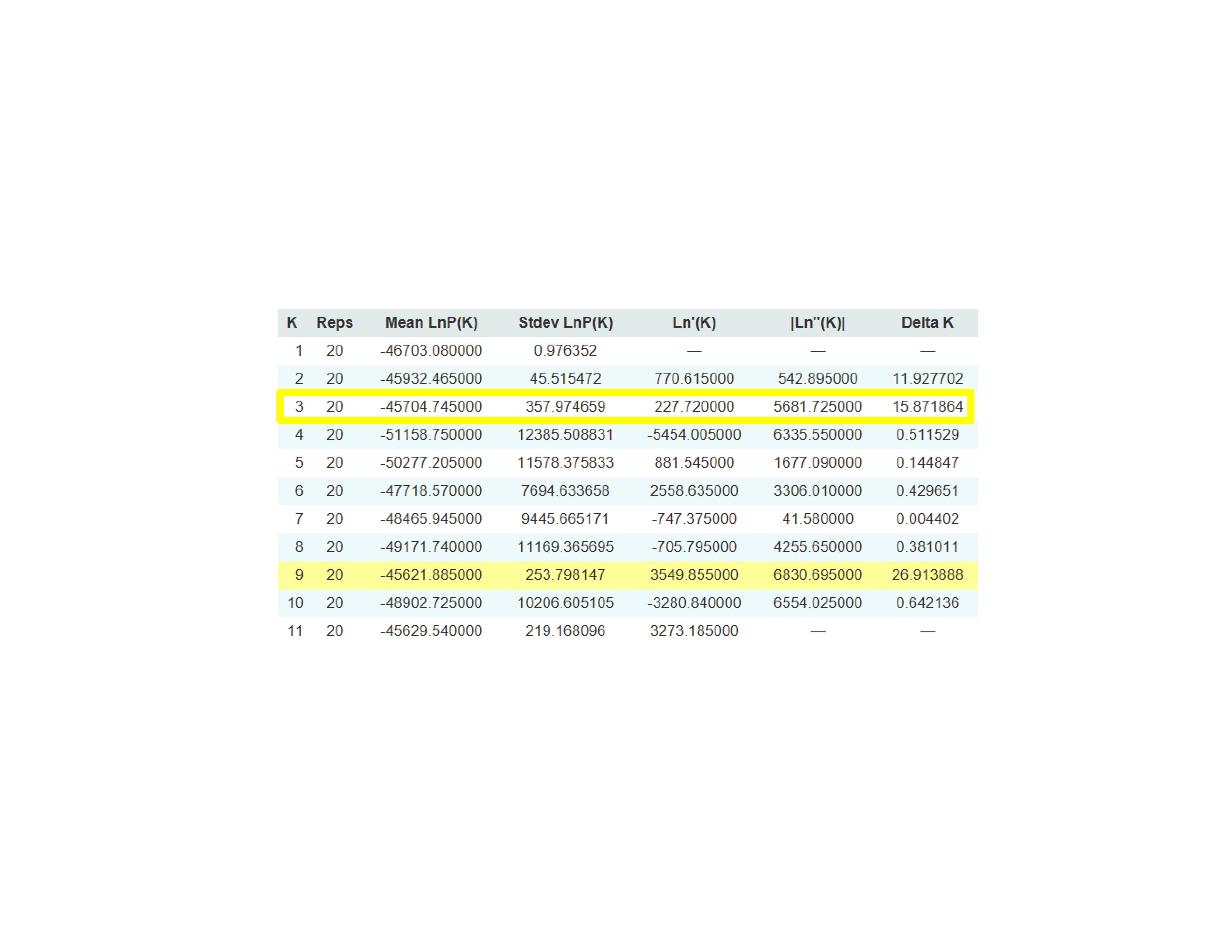

Supplement: Supplementary file 1 — Results from a Structure Harvester analysis of all eight populations of the Cx. pipiens complex in this study. Each row shows the probability of K populations and delta K. The most likely number of populations was K = 3. (TIFF 2681 kb) [file 13071_2017_2594_MOESM1_ESM.tiff]

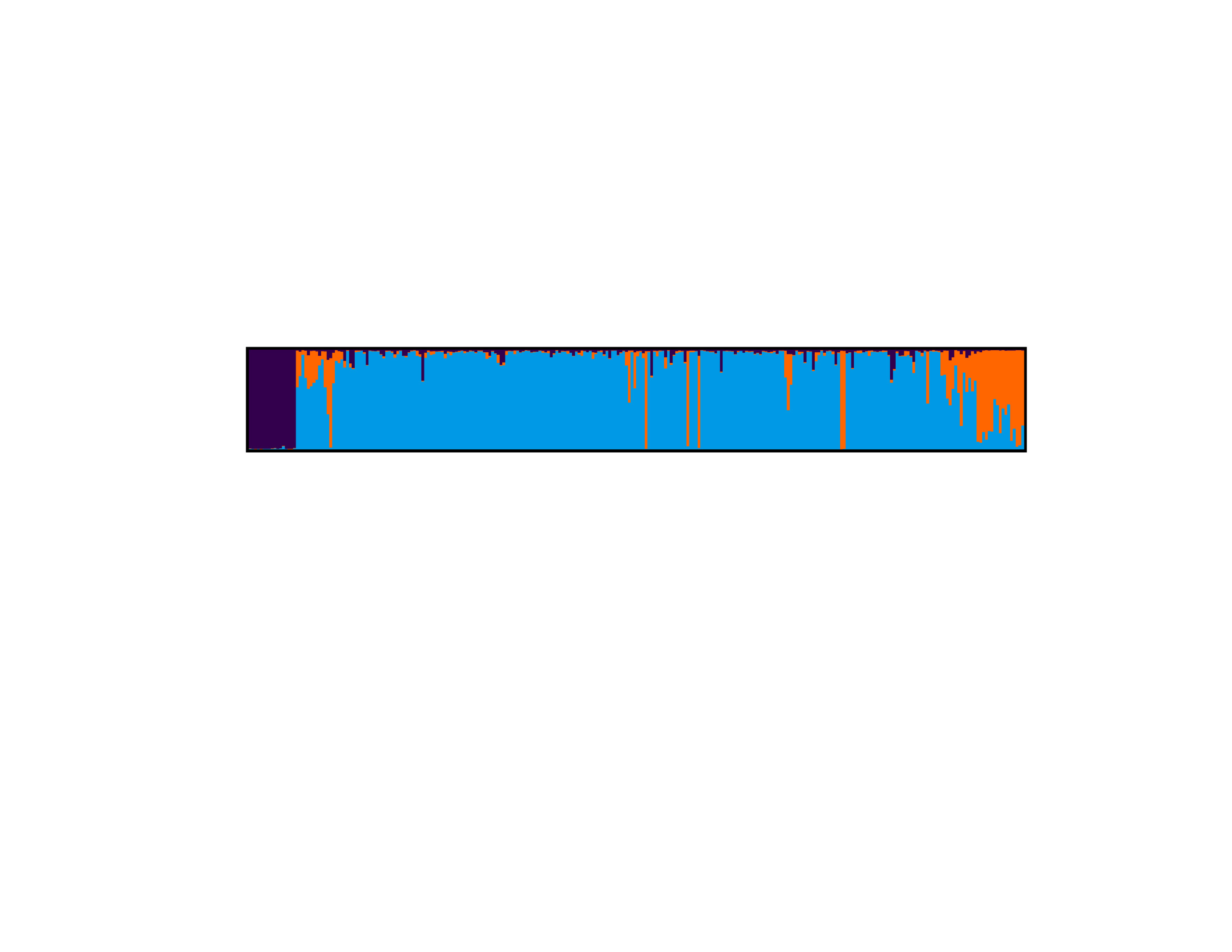

Supplement: Supplementary file 2 — The results from Distruct using output from Structure for K = 3 populations. (TIFF 2317 kb) [file 13071_2017_2594_MOESM2_ESM.tiff]

***Cx. quinquefasciatus***

**D1**

**D2**

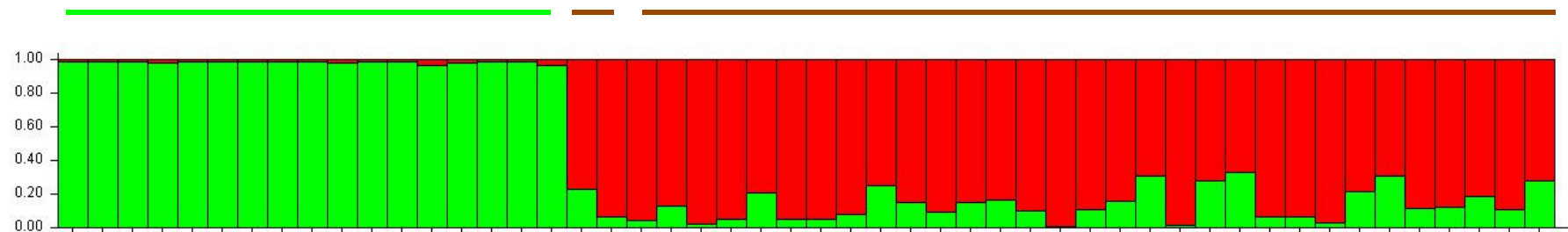

**D2**

**D3**

**D4**

**D5**

***Cx. pipiens***

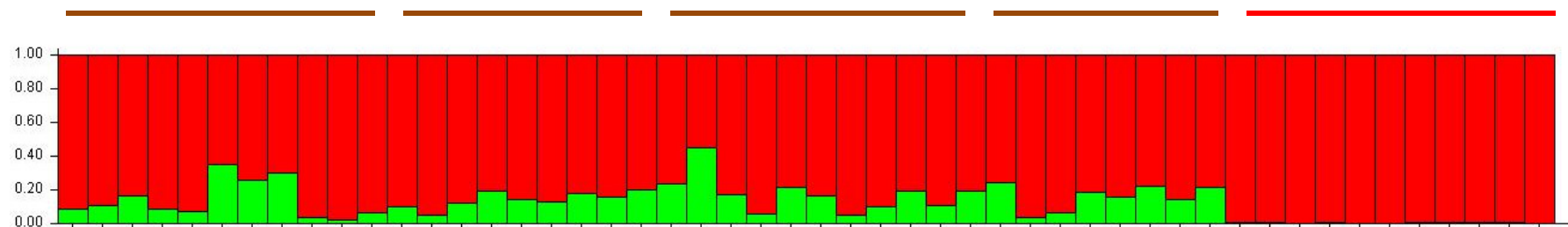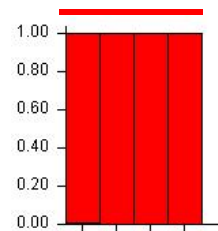

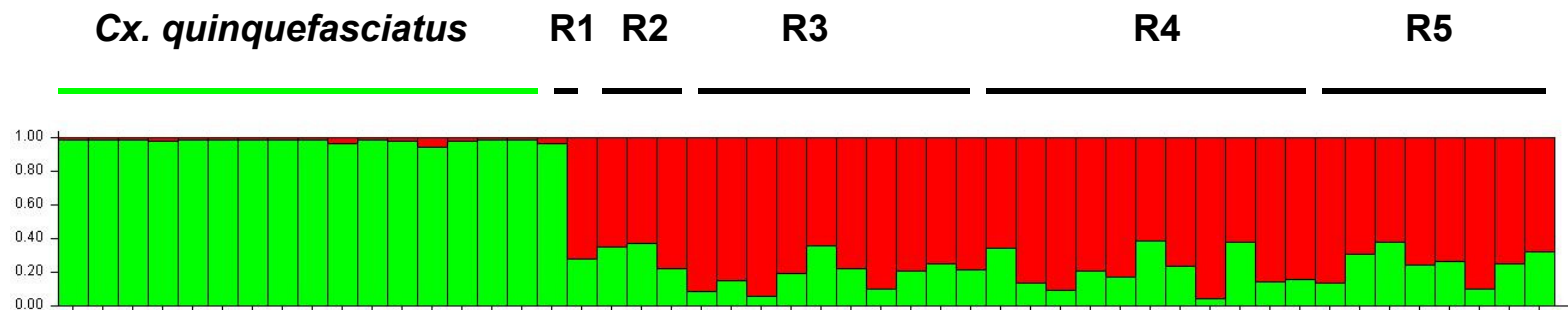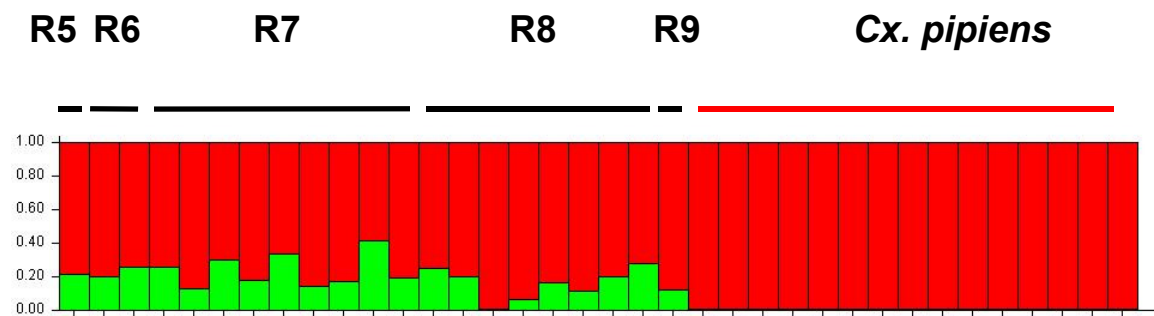

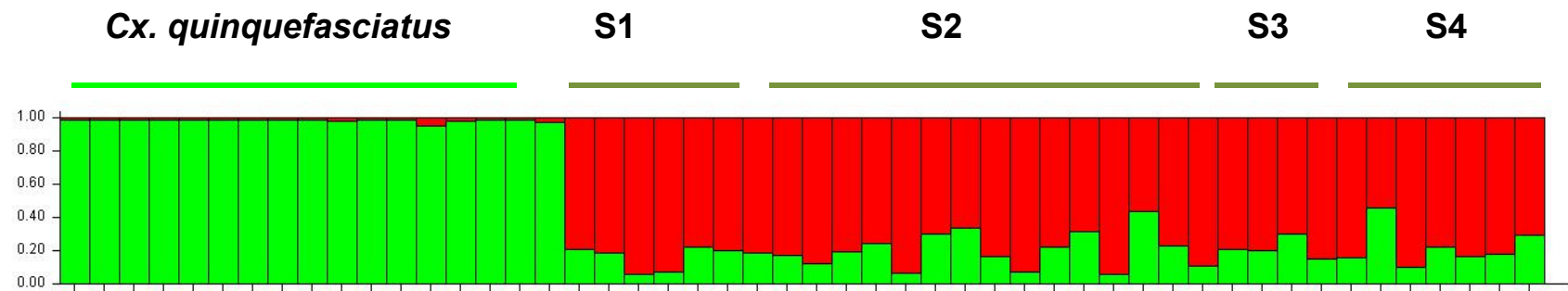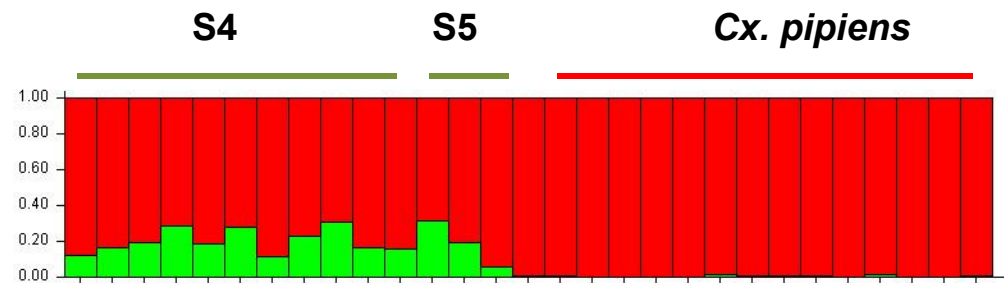

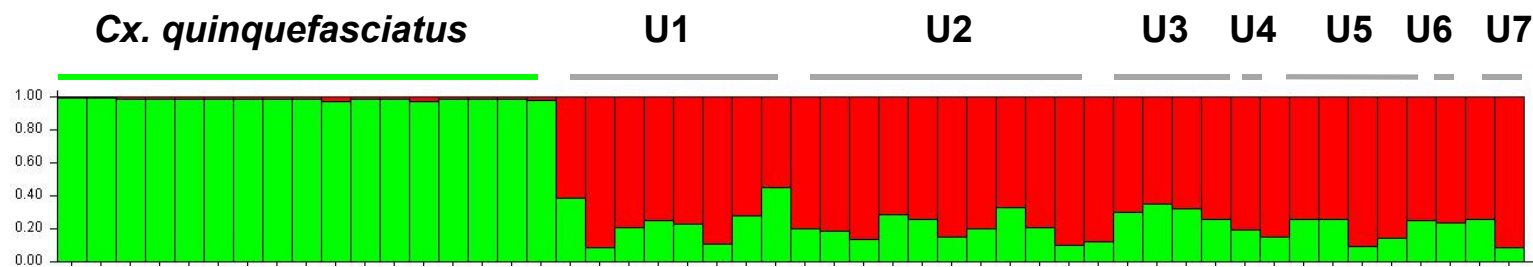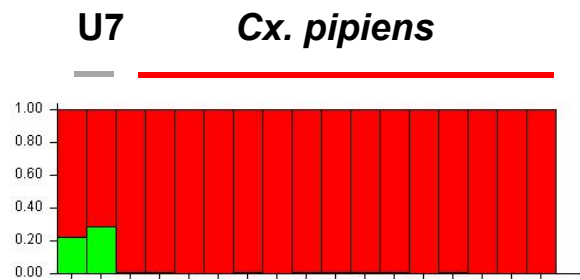

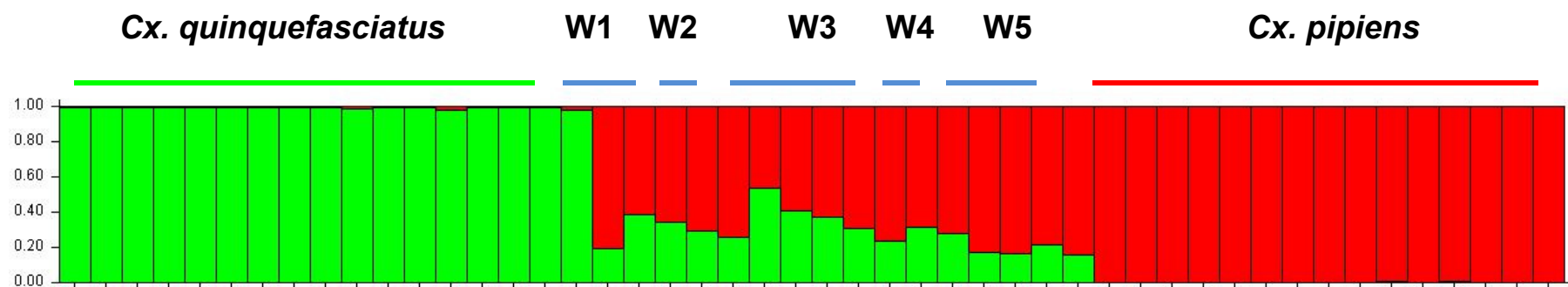

***Cx. quinquefasciatus***

**Oroville**

***Cx. pipiens***

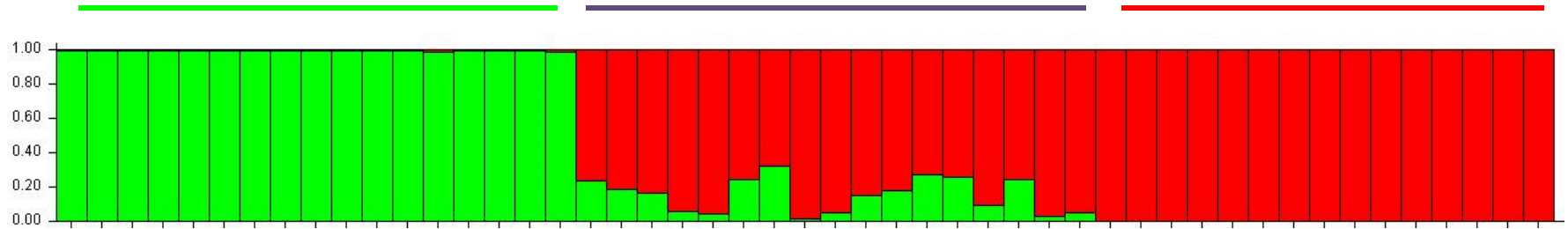

Supplement: Supplementary file 3 — Structure analyses for each of five Merced habitat-associated populations and for the Oroville California population individually compared to Cx. quinquefasciatus from Coachella, California and to Cx. pipiens from Washington. Structure was run using the following parameters: diploid individuals, 100,000 iterations, admixed data, and independent loci. Each vertical bar represents an individual mosquito. Structure Harvester found K = 2 clusters. The y-axis shows the probability of an individual being assigned to one of the two genetic clusters. Panels include a dairy (D) collections, b rural (R) habitat collections, c suburban (S) collections, d urban (U) collections, e wetland (W) collections, and f Oroville California, each compared to pure Cx. quinquefasciatus and Cx. pipiens populations. (PDF 659 kb) [file 13071_2017_2594_MOESM3_ESM.pdf]
